# Critical phenomenon of the near room temperature skyrmion material FeGe

**DOI:** 10.1038/srep22397

**Published:** 2016-02-29

**Authors:** Lei Zhang, Hui Han, Min Ge, Haifeng Du, Chiming Jin, Wensen Wei, Jiyu Fan, Changjin Zhang, Li Pi, Yuheng Zhang

**Affiliations:** 1 High Magnetic Field Laboratory, Chinese Academy of Sciences, Hefei 230031, China; 2 University of Science and Technology of China, Hefei 230026, China; 3 Hefei National Laboratory for Physical Sciences at the Microscale, University of Science and Technology of China, Hefei 230026, China; 4 Department of Applied Physics, Nanjing University of Aeronautics and Astronautics, Nanjing 210016, China

## Abstract

The cubic B20 compound FeGe, which exhibits a near room temperature skyrmion phase, is of great importance not only for fundamental physics such as nonlinear magnetic ordering and solitons but also for future application of skyrmion states in spintronics. In this work, the critical behavior of the cubic FeGe is investigated by means of bulk dc-magnetization. We obtain the critical exponents (*β* = 0.336 ± 0.004, *γ* = 1.352 ± 0.003 and *β* = 5.276 ± 0.001), where the self-consistency and reliability are verified by the Widom scaling law and scaling equations. The magnetic exchange distance is found to decay as 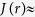 r^−4.9^, which is close to the theoretical prediction of 3D-Heisenberg model (r^−5^). The critical behavior of FeGe indicates a short-range magnetic interaction. Meanwhile, the critical exponents also imply an anisotropic magnetic coupling in this system.

In recently years, skyrmion state, which is a topologically protected nanoscale vortex-like spin structure, has attracted great interest due to its potential application in spintronic storage function^1^,^2^,^3^,^4^,^5^,^6^,^7^,^8^,^9^,^10^,^11^,^12^. It has been demonstrated that the skyrmion phase is thermodynamically stable magnetic vortex state in magnetic crystals^13^,^14^. In addition, writing and deleting single magnetic skyrmion have been realized in PdFe bilayer on Ir(111) surface^15^,^16^. These findings pave a significant path to design quantum-effect devices based on the tunable skyrmion dynamics. The room-temperature skyrmion materials hosting stable skrymion phase are paid considerable attention^17^. The cubic FeGe belongs to the space group 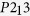, in which the non-centrosymmetric cell results in a weak Dzyaloshinskii-Moriya (DM) interaction. The competition of DM interaction between the much stronger ferromagnetic exchange finally causes a long modulation period of a helimagnetic ground state^1^,^2^,^18^. A bulk FeGe sample exhibits a long-range magnetic order at Curie temperature *T*_*C*_ = 278.2 K, and displays a complex succession of temperature-driven crossovers in the vicinity of *T*_*C*_^19^,^20^. The skyrmion phase emerges in a narrow temperature range just below *T*_*C*_ in the filed range from 0.15 to 0.4 kOe. The existence of the near room temperature skyrmion phase in FeGe, to our knowledge the highest *T*_*C*_ in B20 skyrmion compounds, makes it one of the most promising candidate of the next generation spintronic devices. Recently, more stable skyrmion phase has been realized in FeGe thin film, and it has been claimed that the skyrmions can be tuned by the crystal lattice^21^,^22^,^23^. On the other hand, multiple and complex magnetic interactions have also been found in FeGe. An inhomogeneous helimagnetic state has been discovered above *T*_*C*_ due to the strong precursor phenomena^19^,^24^. More interestingly, it has been revealed that the helical axis (*q*-vector direction) orientates depending on temperature. At zero magnetic field, the helical axis is along the 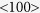 direction below 280 K. With decreasing temperature, it changes to the 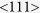 direction at 211 K^20^.

In view of the potential application and abundant physics in FeGe, a deep investigation of its magnetic exchange is of great importance not only for fundamental physics such as nonlinear magnetic ordering and solitons but also for creation of a basic for future application of skyrmion states and other chiral modulations in spintronics. In this work, the critical behavior of FeGe has been investigated by means of bulk dc-magnetization. The critical exponents (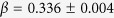, 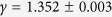, and 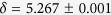) are obtained, where the self-consistency and reliability are verified by the Widom scaling law and the scaling equations. These critical behavior of FeGe indicates a short-range magnetic interaction with a magnetic exchange distance decaying as 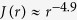. The obtained critical exponents also imply an anisotropic magnetic coupling in FeGe system.

## Results and Discussion

It is well known that the critical behavior for a second-order phase transition can be investigated through a series of critical exponents. In the vicinity of the critical point, the divergence of correlation length 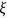 leads to universal scaling laws for the spontaneous magnetization *M*_*S*_ and initial susceptibility 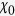. Subsequently, the mathematical definitions of the exponents from magnetization are described as^25^,^26^:


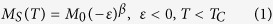



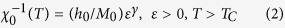



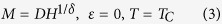


where 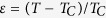 is the reduced temperature; 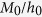 and *D* are the critical amplitudes. The parameters *β* (associated with 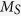), *γ* (associated with 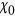), and 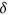 (associated with *T*_*C*_) are the critical exponents. Universally, in the asymptotic critical region 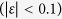, these critical exponents should follow the Arrott-Noakes equation of state^27^:


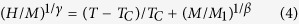


Therefore, the critical exponents *β* and *γ* can be obtained by fitting the 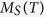 and 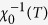 curves using the modified Arrott plot of 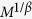 vs 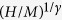. Meanwhile, *δ* can be generated directly by the 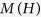 at the critical temperature *T*_*C*_ according to Eq. (3).

**Figure 1:**
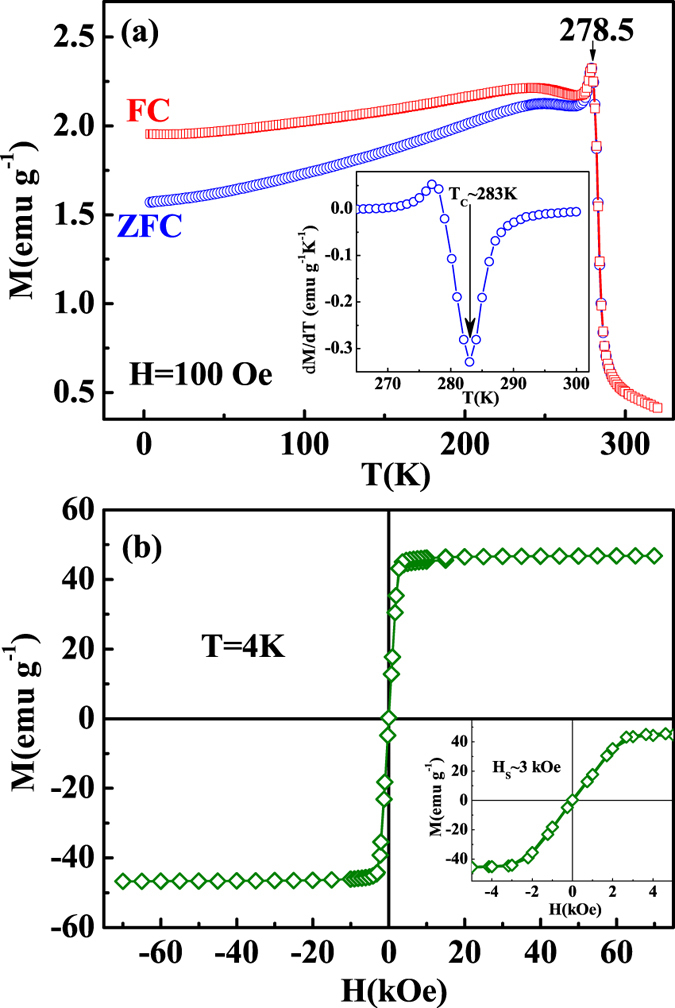
(**a**) The temperature dependence of magnetization [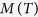] for FeGe under *H *= 100 Oe [the inset shows the derivative magnetization (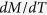) vs *T*]; (**b**) the isothermal magnetization 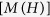 at 4 K (the inset gives the magnified region in the lower field regime).

Generally, the critical temperature *T*_*C*_ can be roughly determined by the temperature dependence of magnetization [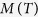]. Figure 1(a) shows the 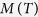 curves for FeGe under zero-field-cooling (ZFC) and field-cooling (FC) with an applied field *H *= 100 Oe. The 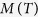 curves exhibit an abrupt decline with the increase of temperature, corresponding to the paramagnetic-helimagnetic (PM-HM) transition. A sharp peak is observed at *T* = 278.5 K. The inset of Fig. 1(a) gives 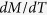 vs *T*, where *T*_*C*_ ≈ 283 K is determined from the minimum of the 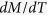 curve. Wilhelm *et al*. has demonstrated that a long-rang magnetic order occurs below 278.2 K, however, an inhomogeneous helical state has existed above that temperature due to the strong precursor phenomena^19^,^28^. The higher *T*_*C*_ determined here indicates the appearance of precursor phenomena which may be caused by the strong spin fluctuation^24^. Figure 1(b) shows the isothermal magnetization 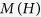 at 4 K, which exhibits a typical magnetic ordering behavior. The inset of Fig. 1(b) plot the magnified 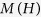 in lower field regime, which shows that the saturation field *H*_*S*_ ≈ 3000 Oe. No magnetic hysteresis is found on the 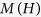 curve, indicating no coercive force for FeGe.

**Figure 2:**
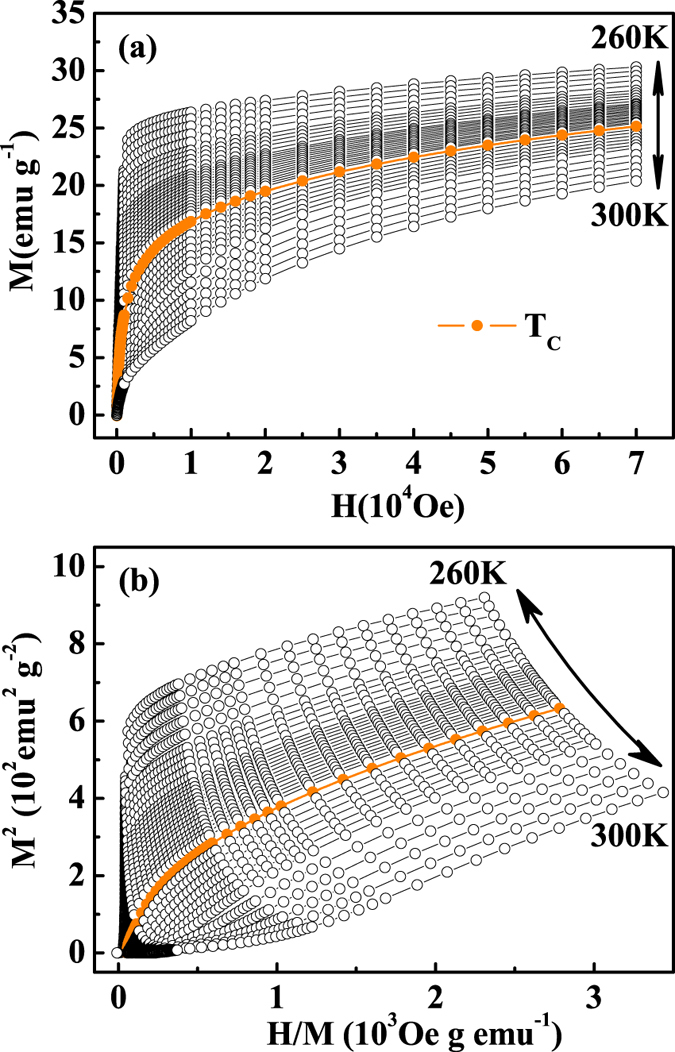
(**a**) The initial magnetization around 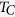 for FeGe; (**b**) Arrott plots of *M*^2^ vs 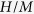 [the 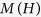 curves are measured at interval 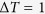 K, and 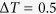 K when approaching *T*_*C*_].

Usually, the critical exponents can be determined by the Arrott plot. For the Landau mean-field model with 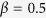 and 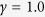
29, the Arrott-Noakes equation of state evolves into 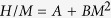, the so called Arrott equation. In order to construct an Arrot plot, the isothermal magnetization curves 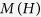 around *T*_*C*_ are measured as shown in Fig. 2(a). The Arrott plot of *M*^2^ vs 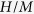 for FeGe is depicted in Fig. 2(b). According to the Banerjee’s criterion, the slope of line in the Arrott plot indicates the order of the phase transition: negative slope corresponds to first-order transition while positive to second-order one^30^. Therefore, the Arrott plot of FeGe implies a second-order phase transition, in agreement with the specific heat measurement^28^. According to the Arrott plot, the *M*^2^ vs 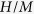 generally present a series of parallel straight lines around *T*_*C*_, where 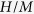 vs. *M*^2^ at *T*_*C*_ just pass through the origin^31^. One can see that all *M*^2^ vs 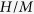 curves show quasi-straight lines with positive slopes in high field range. However, all lines show an upward curvature and are not parallel to each other, indicating that the 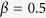 and 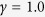 within the framework of Landau mean-field model is unsatisfied. Therefore, a modified Arrott plot should be employed.

**Figure 3:**
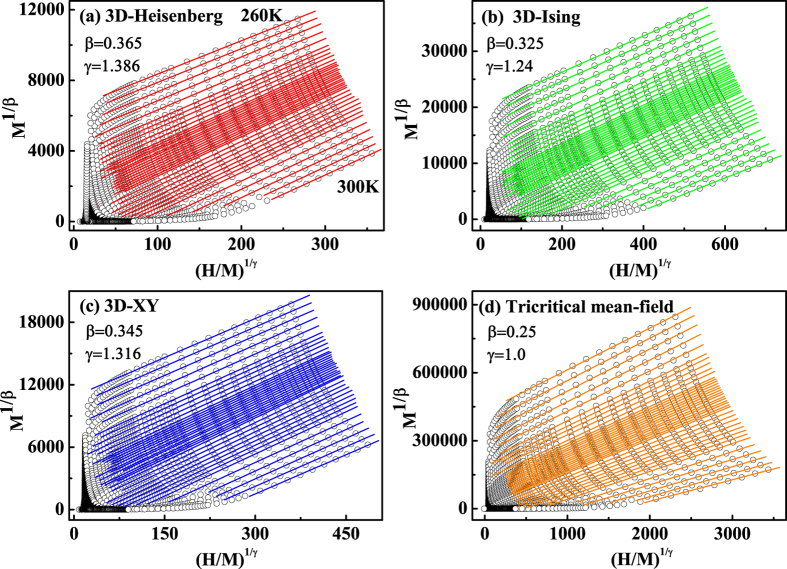
The isotherms of 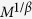 vs 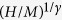 with (**a**) 3D-Heisenberg model; (**b**) 3D-Ising model; (**c**) 3D-XY model; and (**d**) tricritical mean-field model.

**Figure 4:**
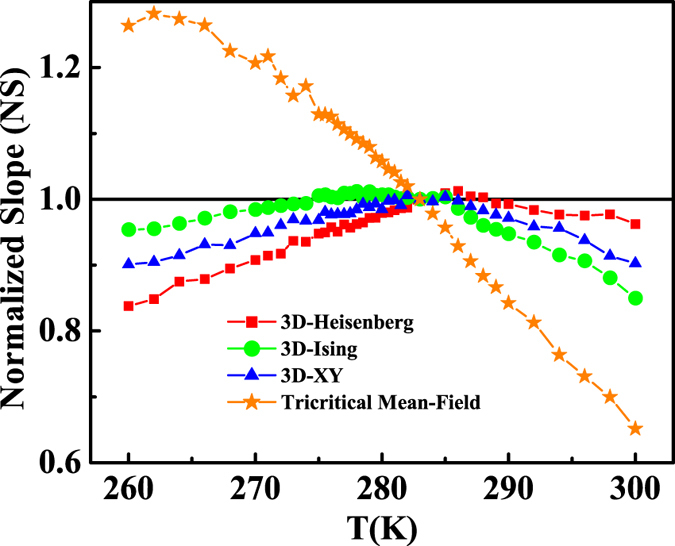
The normalized slopes [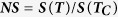] as a function of temperature.

Four kinds of possible exponents belonging to the 3D-Heisenberg model 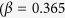, 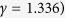, 3D-Ising model 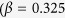, 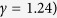, 3D-XY model 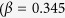, 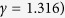, and tricritical mean-field model 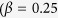, 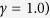
29,32 are used to construct the modified Arrott plots, as shown in Fig. 3 (a–d). All these four constructions exhibit quasi-straight lines in the high field region^33^,^34^,^35^. Apparently, the lines in Fig. 3(d) are not parallel to each other, indicating that the tricritical mean-field model is not satisfied. However, all lines in Fig. 3(a–c) are almost parallel to each other. To determine an appropriate model, the modified Arrott plots should be a series of parallel lines in the high field region with the same slope, where the slope is defined as 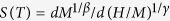. The normalized slope (*NS*) is defined as 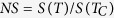, which enables us to identify the most suitable model by comparing the *NS* with the ideal value of ‘1’^33^. Plots of *NS* vs *T* for the four different models are shown in Fig. 4. One can see that the *NS* of 3D-Heisenberg model is close to ‘1’ mostly above *T*_*C*_, while that of 3D-Ising model is the best below *T*_*C*_. This result indicates that the critical behavior of FeGe may not belong to a single universality class.

**Figure 5:**
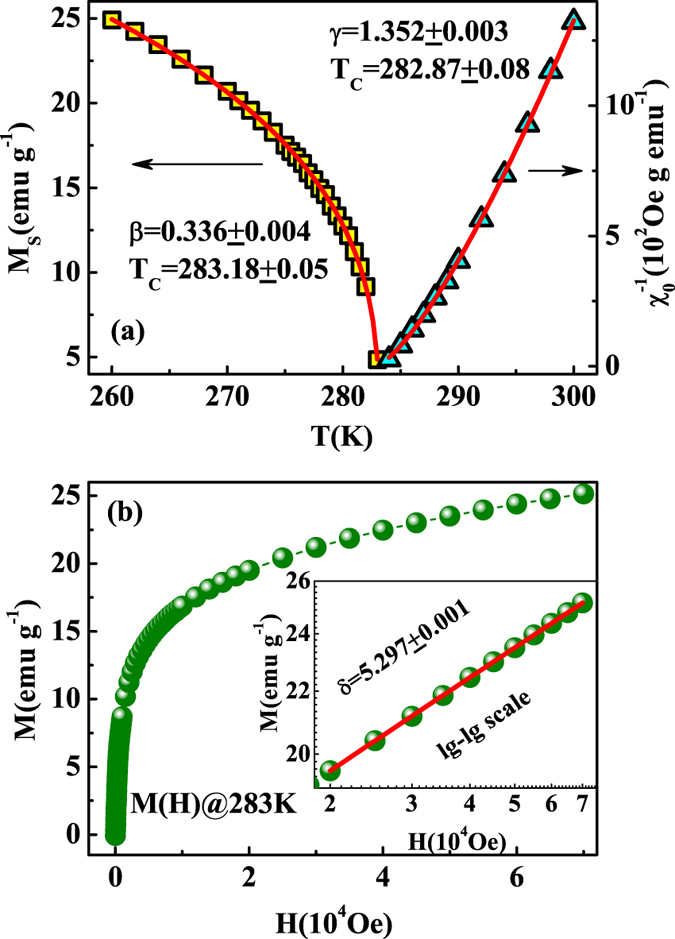
(**a**) The temperature dependence of 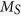 and χ_0_^−1^ for FeGe with the fitting solid curves; (**b**) the isothermal 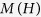 at *T*_*C*_ with the inset plane on 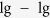 scale (the solid line is fitted).

The precise critical exponents *β* and *γ* should be achieved by the iteration method^36^. The linear extrapolation from the high field region to the intercepts with the axes 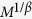 and 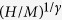 yields reliable values of 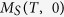 and 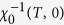, which are plotted as a function of temperature in Fig. 5(a). By fitting to Eqs. (1) and (2), one obtains a set of *β* and *γ*. The obtained *β* and *γ* are used to reconstruct a new modified Arrott plot. Consequently, new 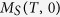 and 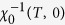 are generated from the linear extrapolation from the high field region. Therefore, another set of *β* and *γ* can be yielded. This procedure is repeated until *β* and *γ* do not change. As one can see, the obtained critical exponents by this method are independent on the initial parameters, which confirms these critical exponents are reliable and intrinsic. In this way, it is obtained that 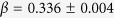 with 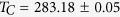 and 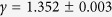 with 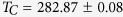 for FeGe. The critical temperature *T*_*C*_ from the modified Arrott plot is in agreement with that obtained from the derivative 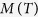 curve, indicating strong critical fluctuation before the formation of the long-range ordering in FeGe^24^. This critical fluctuation is in agreement with the precursor phenomenon reported by Wilhelm *et al*.^28^. The modulated precursor states and complexity of the magnetic phase diagram near the magnetic ordering are explained by the change of the character of solitonic inter-core interactions and the onset of specific confined chiral modulations^19^,^28^.

Figure 5(b) shows the isothermal magnetization 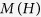 at the critical temperature 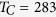 K, with the inset plotted on a 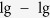 scale. One can see that the 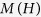 at *T*_*C*_ exhibits a straight line on a 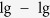 scale for 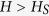. We determine that 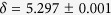 in the high field region 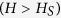. According to the statistical theory, these critical exponents should fulfill the Widom scaling law^37^:


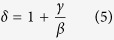


As a result, 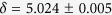 is calculated according to the Widom scaling law, in agreement with the results from the experimental critical isothermal analysis. The self-consistency of the critical exponents demonstrates that they are reliable and unambiguous.

**Figure 6:**
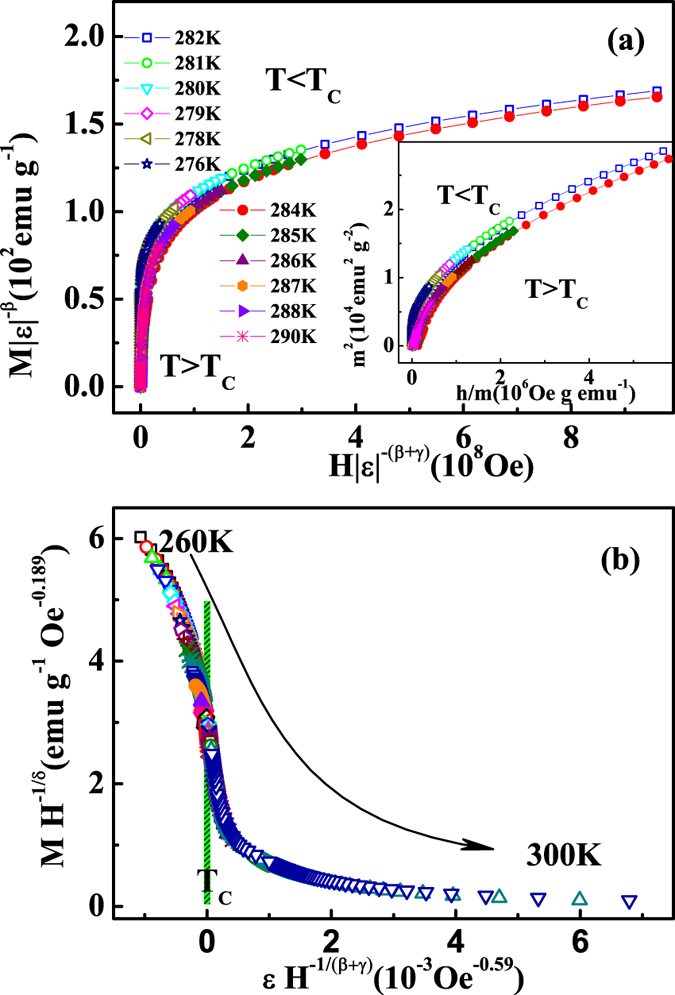
(**a**) Scaling plots of renormalized magnetization *m* vs renormalized field *h* around the critical temperatures for FeGe (the inset shows the *m*^2^ vs 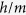); (**b**) the rescaling of the the 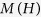 curves by 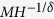 vs 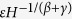.

Finally, these critical exponents should obey the scaling equations. Two different constructions have been used in this work, both of which are based on the scaling equations of state. According to the scaling equations, in the asymptotic critical region, the magnetic equation is written as^25^:


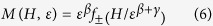


where 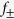 are regular functions denoted as 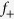 for 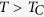 and 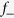 for 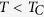. Defining the renormalized magnetization as 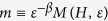, and the renormalized field as 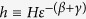, the scaling equation indicates that *m* vs *h* forms two universal curves for 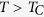 and 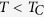 respectively^38^,^39^. Based on the scaling equation 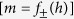, the isothermal magnetization around *T*_*C*_ for FeGe is replotted in Fig. 6(a), where all experimental data collapse onto two universal branches. The inset of Fig. 6(a) shows he *m*^2^ vs 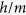, where all 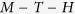 curves should collapse onto two independent universal curves. In addition, the scaling equation of state takes another form^25^,^38^:


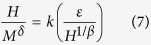


where 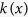 is the scaling function. Based on Eq. (7), all experimental curves will collapse onto a single curve. Figure 6(b) shows the 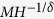 vs 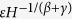 for FeGe, where the experimental data collapse onto a single curve, and *T*_*C*_ locates at the zero point of the horizontal axis. The well-rescaled curves further confirm the reliability of the obtained critical exponents.

The obtained critical exponents of FeGe and other related materials, as well as those from different theoretical models are summarized in Table 1 for comparison. One can see that the critical exponent *γ* of FeGe is close to that of 3D-Heisenberg model, while *β* approaches to that of 3D-Ising or 3D-XY mode, indicating that the critical behavior of FeGe do not belong to a single universality class. Anyhow, all these three models indicate a short-range magnetic coupling, implying the existence of short-range magnetic interaction in FeGe. As we know, for a homogeneous magnet, the universality class of the magnetic phase transition depends on the exchange distance 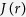. M. E. Fisher *et al*. have treated this kind of magnetic ordering as an attractive interaction of spins, where a renormalization group theory analysis suggests 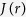 decays with distance *r* as^40^,^41^:


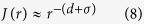


where *d* is the spatial dimensionality and *σ* is a positive constant. Moreover, there is^41^,^42^:


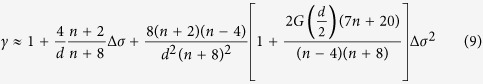


where 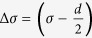 and 
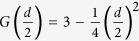
, *n* is the spin dimensionality. For a three dimensional material (*d* = 3), we have 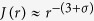. When 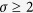, the Heisenberg model (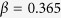, 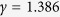 and 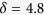) is valid for the three dimensional isotropic magnet, where 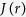 decreases faster than *r*^−5^. When 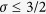, the mean-field model 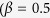, 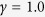 and 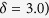 is satisfied, expecting that 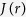 decreases slower than 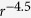. From Eq. (9) 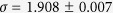 is generated for FeGe, thus close to the short-range magnetic coupling of 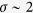. Subsequently, it is found that the magnetic exchange distance decays as 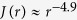, which indicates that the magnetic coupling in FeGe is close to a short-range interaction. Moreover, we get the correlation length critical exponent 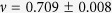 (where 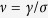, 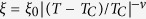), and 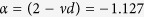 ± 0.008. Theory gives that 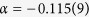 for 3D-Heisenberg model and 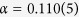 for 3D-Ising model^43^,^44^. Therefore, these critical exponents indicates that the critical behavior in FeGe is close to the 3D-Heisenberg model with short-range magnetic coupling. However, the discrepancy of the critical exponents to 3D-Ising or 3D-XY models indicates an anisotropic magnetic exchange interaction.

As can be seen from Table 1, the critical exponents of Fe_0.8_Co_0.2_Si and Cu_2_OSeO_3_, which also exhibit a helimagnetic and skyrmion phase transition with similar crystal symmetry, are close to the universality class of the 3D-Heisenberg model^45^,^46^, indicating a isotropic short-range magnetic coupling. However, the critical behavior of MnSi belongs to the tricritical mean field model^47^,^48^. In macroscopic view, the magnetic ordering in cubic FeGe is a DM spiral similar to the structure observed in the isostructural compound MnSi^49^. However, in microscopic view, the magnetic coupling types in these two helimagnets are different. The critical behavior of FeGe is roughly similar to those of Fe_0.8_Co_0.2_Si or Cu_2_OSeO_3_, except a magnetic exchange anisotropy. In MnSi the spiral propagates are along equivalent 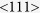 directions at all temperatures below 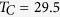 K. However, it has been revealed that the helical axis (*q*−vector direction) in FeGe depends on temperature. It is along the 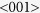 direction below 280 K, and changes to the 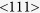 direction in a lower temperature range at 211 K with the decrease of temperature at zero magnetic field^20^. This unique change of helical axis in FeGe may be correlated with the anisotropy of magnetic exchange in this system, since the magnetic exchange anisotropy also plays an important role in determination of the spin ordering direction. In addition, it should be expounded that the magnetic exchange anisotropy is essentially different from the magnetocrystalline anisotropy. The magnetocrystalline anisotropy is correlated to the crystal structure, while magnetic exchange anisotropy originates from the anisotropic magnetic exchange coupling 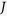.

## Conclusion

In summary, the critical behavior of the near room temperature skyrmion material FeGe has been investigated around *T*_*C*_. The reliable critical exponents (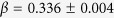, 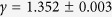, and 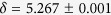) are obtained, which are verified by the Widom scaling law and scaling equations. The magnetic exchange distance is found to decay as 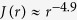, which is close to that of 3D-Heisenberg model (*r*^−5^). The critical behavior indicate that the magnetic interaction in FeGe is of short-range type with an anisotropic magnetic exchange coupling.

## Methods

A polycrystalline B20-type FeGe sample was synthesized with a cubic anvil-type high-pressure apparatus. The detailed preparing method was described elsewhere, and the physical properties were carefully checked [H. Du. *et al*., Nat. Commun. **6**, 8504 (2015)]. The chemical compositions were determined by the Energy Dispersive X-ray (EDX) Spectrometry as shown in Fig. S1 and Table SI, which shows the atomic ratio of Fe : Ge ≈ 50.52: 49.48. The magnetization was measured using a Quantum Design Vibrating Sample Magnetometer (SQUID-VSM). The no-overshoot mode was applied to ensure a precise magnetic field. To minimize the demagnetizating field, the sample was processed into slender ellipsoid shape and the magnetic field was applied along the longest axis. In addition, the isothermal magnetization was performed after the sample was heated well above 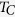 for 10 minutes and then cooled under zero field to the target temperatures to make sure curves were initially magnetized. The magnetic background was carefully subtracted. The applied magnetic field 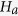 has been corrected into the internal field as 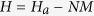 (where *M* is the measured magnetization and *N* is the demagnetization factor) [A. K. Pramanik *et al*., Phys. Rev. B **79**, 214426 (2009)]. The corrected *H* was used for the analysis of critical behavior.

## Additional Information

**How to cite this article**: Zhang, L. *et al*. Critical phenomenon of the near room temperature skyrmion material FeGe. *Sci. Rep.* **6**, 22397; doi: 10.1038/srep22397 (2016).

## Supplementary Material

Supplementary Information

## Figures and Tables

**Table 1:** Comparison of critical exponents of FeGe with different theoretical models and related materials (MAP = modified Arrott plot; Hall = Hall effect; AC = ac susceptibility; SC = single crystal; PC = polycrystal).

Composition	technique	Ref.	*T*_*C*_(K)	*β*	*γ*	*δ*
FeGe^*PC*^	MAP	This work	283	0.336 ± 0.004	1.352 ± 0.003	5.267 ± 0.001
3D-Heisenberg	theory	29	–	0.365	1.386	4.8
3D-XY	theory	29	–	0.346	1.316	4.81
3D-Ising	theory	29	–	0.325	1.24	4.82
Tricritical mean-field	theory	32	–	0.25	1.0	5.0
Mean-field	theory	29	–	0.5	1.0	3.0
MnSi^*SC*^	MAP	48	30.5	0.242 ± 0.006	0.915 ± 0.003	4.734 ± 0.006
Fe_0.8_Co_0.2_Si^*PC*^	Hall	45	36.0	0.371 ± 0.001	1.38 ± 0.002	4.78 ± 0.01
Cu_2_OSeO_3_^*SC*^	AC	46	58.3	0.37(1)	1.44(4)	4.9(1)
