# Supplementary material for: Critical phenomenon of the near room temperature skyrmion material FeGe
Source: Sci Rep. 2016 Feb 29;6:22397. doi: 10.1038/srep22397 (PMC4772635; doi:10.1038/srep22397)
Supplement: Supplementary Information [file srep22397-s1.pdf]

# Critical phenomenon of the near room temperature skyrmion material FeGe

Lei Zhang,[1,*] Hui Han,[1,2] Min Ge,[3] Haifeng Du,[1] Chiming Jin,[1] Wensen Wei,[1] Jiyu Fan,[4] Changjin Zhang,[1] Li Pi,[1,3] and Yuheng Zhang[1,3]

[1]*High Magnetic Field Laboratory, Hefei Science Center, Chinese Academy of Sciences, Hefei 230031, China*

[2]*University of Science and Technology of China, Hefei 230026, China*

[3]*Hefei National Laboratory for Physical Sciences at the Microscale, University of Science and Technology of China, Hefei 230026, China*

[4]*Department of Applied Physics, Nanjing University of Aeronautics and Astronautics, Nanjing 210016, China*

(Dated: November 20, 2015)

PACS numbers: 75.40.-s, 75.40.Cx, 75.40.Gb

Keywords: helimagnetism; critical behavior; 3D-Heiseberg model, short-range magnetic coupling

*Corresponding author. Email: zhanglei@hmfl.ac.cn

## I. SAMPLE PREPARATION

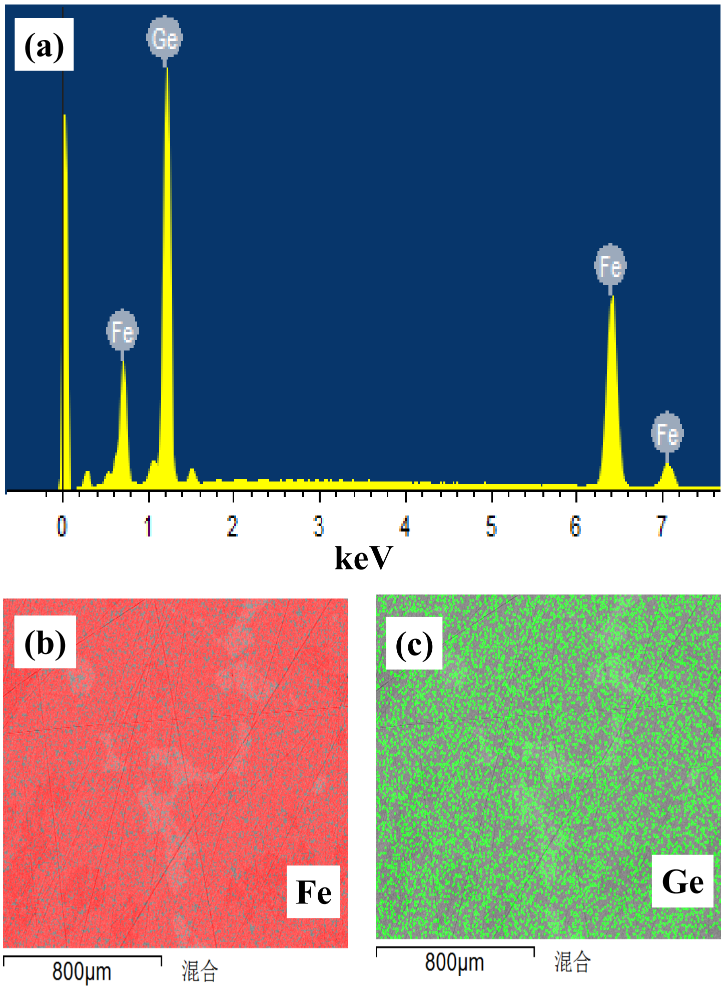


Fig. S 1: (Color online) (a) The Energy Dispersive X-ray (EDX) spectrum for FeGe; the elemental distribution of Fe (b) and Ge (c).

A polycrystalline B20-type FeGe sample was synthesized with a cubic anvil-type high-pressure apparatus. A mixture of the elemental materials with an atomic ratio of 1:1 was synthesized by electromagnetic induction melting in an argon atmosphere. The alloy was placed into a cylindrical BN capsule and was heat-treated for 1 h at 1073 K under a high pressure of 4 GPa. The detailed preparing method was described elsewhere, and the physical properties were carefully checked [1].

Table S I: Elemental ratio of Fe and Ge in FeGe sample determined by the EDX spectrometry.

| Element | weight ratio (%) | atomic ratio (%) |
|---|---|---|
| Fe $K$ | 43.99 | 50.52 |
| Ge $K$ | 56.01 | 49.48 |
| total | 100% | |

## II. EXPERIMENT AND FITTING DETAILS

The magnetization was measured using a Quantum Design vibrating sample magnetometer (SQUID-VSM). The no-overshoot mode was applied to ensure a precise magnetic field. In addition, the field was relaxed for 2 minutes before the data collection. For the measurement the sample was processed into slender ellipsoid shape, and the magnetic field was applied along the longest axis to minimize the demagnetizating field. To make sure each curve was initially magnetized, the isothermal magnetization was performed after the sample was heated well above $T_C$ for 10 minutes and then cooled under zero field to the target temperatures. The magnetic background was carefully subtracted. The applied magnetic field $H_a$ has been corrected into the internal field as $H = H_a - NM$ (where $M$ is the measured magnetization and $N$ is the demagnetization factor) [2]. The corrected $H$ was used for the analysis of critical behavior.

The chemical compositions of the used FeGe are determined from the Energy Dispersive X-ray (EDX) Spectrometry as shown in Fig. S1 (a) and Table S I, which shows the atomic ratio of Fe : Ge $\approx$ 50.52 : 49.48. The elemental distribution of Fe and Ge are shown in Fig S1 (b) and (c) respectively, indicating good homogeneity of the sample.

To determine an appropriate model, the modified Arrott plots should be a series of parallel lines in the higher field region with the same slope, where the slope is defined as $S(T) = dM^{1/\beta}/d(H/M)^{1/\gamma}$. The normalized slope ($NS$) is defined as $NS = S(T)/S(T_C)$, as shown in Fig. S 2, which enables one to identify the most suitable model by comparing

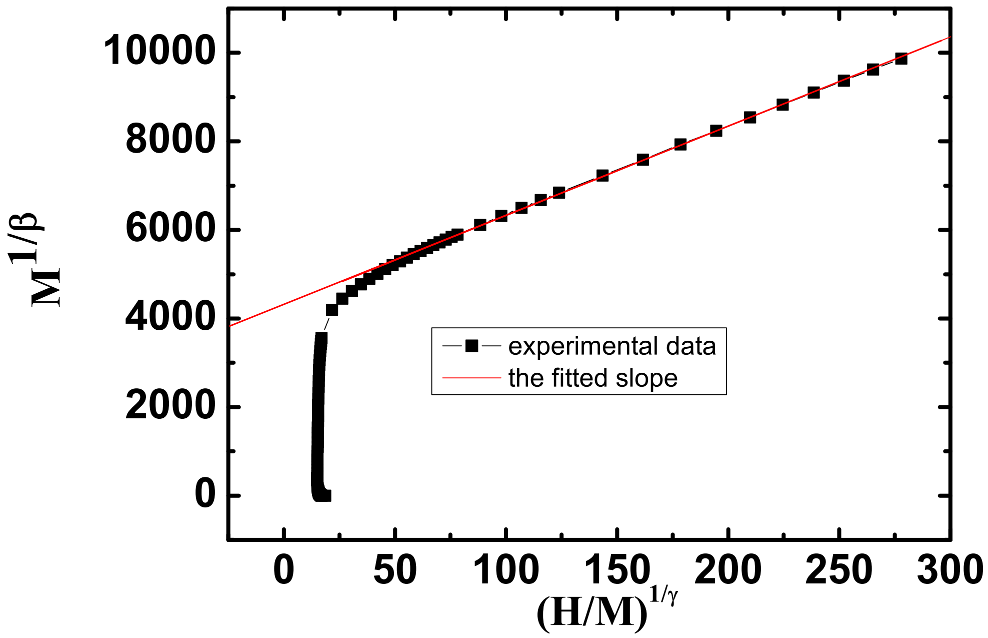


Fig. S 2: (Color online) The obtain of the slope $S(T) = dM^{1/\beta}/d(H/M)^{1/\gamma}$.

$NS$ with the ideal value of '1' [3].

---


[1] H. F. Du, R. C. Che, L. Y. Kong, X. B. Zhao, C. M. Jin, C. Wang, J. Y. Yang, W. Ning, R. W. Li, C. Q. jin, X. H. Chen, J. D. Zang, Y. H. Zhang, M. L. Tian, Nat. Commun. **6,** 8504 (2015).

[2] A. K. Pramanik and A. Banerjee, Phys. Rev. B **79,** 214426 (2009).

[3] J. Y. Fan, L. S. Ling, B. Hong, L. Zhang, L. Pi, Y. H. Zhang, Phys. Rev. B **81,** 144426 (2010).
